# Characterization of Unusual Serogroups of *Neisseria meningitidis*

**DOI:** 10.3390/microorganisms12122528

**Published:** 2024-12-07

**Authors:** Samy Taha, Giulia Fantoni, Eva Hong, Aude Terrade, Oumar Doucoure, Ala-Eddine Deghmane, Muhamed-Kheir Taha

**Affiliations:** 1 Institut Pasteur, Invasive Bacterial Infections, Université Paris Cité, 75015 Paris, France; giulia.fantoni@vismederi.com (G.F.); eva.hong@pasteur.fr (E.H.); aude.terrade@pasteur.fr (A.T.); oumar.doucoure@pasteur.fr (O.D.); ala-eddine.deghmane@pasteur.fr (A.-E.D.); 2 Department of Biotechnology, Chemistry and Pharmacy, University of Siena, 53100 Siena, Italy

**Keywords:** *Neisseria meningitidis*, serogroups, complement, vaccine coverage

## Abstract

Most cases of invasive meningococcal disease (IMD) in Europe are caused by isolates of the *Neisseria meningitidis* serogroups B, C, W, and Y. We aimed to explore cases caused by other unusual serogroups. We retrospectively screened IMD cases in the databases of the National Reference Center for Meningococci and *Haemophilus influnezae* in France between 2014 and 2023. Age, sex, serogroups, and genetic lineage distributions were analyzed. We also measured complement deposition on the bacterial surface and tested coverage by vaccines against serogroup B. Cases due to isolates of serogroups other than B, C, W, and Y represented 1.6% of all 3610 IMD cases during the study period with 59 cases and a median age of 21.5 years of age. The corresponding isolates were non-groupable (26 cases), serogroup X (21 cases), serogroup E (11 cases), and one isolate belonged to serogroup Z. Only a low proportion (7.4%) belonged to the hyperinvasive genetic lineages. Isolates of serogroup E bound a significantly higher amount of complement on their surface and were mainly detected in patients with terminal complement pathway deficiencies. Isolates of these unusual serogroups were shown to be covered by vaccines licensed against meningococci B. Surveillance of these isolates needs to be enhanced.

## 1. Introduction

*Neisseria meningitidis* is a causative agent of invasive meningococcal disease (IMD) characterized by severe illnesses such as meningitis and septicemia. Among the 12 known serogroups of *N. meningitidis* that are defined by the antigenicity of the meningococcal capsule, six—A, B, C, W, X, and Y—are responsible for the vast majority of IMD cases around the globe [1]. Moreover, meningococcal isolates can be classified according to their genotypes into sequence types (ST), which can be clustered into clonal complexes (CC). The sequence data that allow this genotyping can now be extracted from whole genome sequences (WGSs) using molecular tools available on the PUBMLST platform (https://pubmlst.org/ accessed on 18 November 2024). Several CC are considered hyperinvasive and are responsible for most IMD cases, including CC11, CC32, CC41/44, and CC269 [2].

Nevertheless, the distribution of these serogroups is not static; it varies geographically and fluctuates over time due to shifts in population immunity, the introduction of vaccination programs, and localized outbreaks. An illustrative example of this geographic variability is the emergence of serogroup X in Africa over the past decade. While serogroup X is historically rare in Europe, the sub-Saharan region—notably within the “meningitis belt”—has experienced a rising number of *N. meningitidis* X infections since 2006. This emergence has been particularly notable in countries such as Nigeria, Niger and Burkina Faso, where ongoing surveillance has detected significant increases in serogroup X-induced IMD, culminating in substantial outbreaks in 2010 and 2012 [2].

However, beyond these six common serogroups, the less frequent or “rare serogroups” of *N. meningitidis*, including E and Z, non-groupable (NG) isolates, and serogroup X in Europe, have also been sporadically implicated in IMD cases. Such isolates constitute a marginal proportion of overall IMD cases in the general population. The incidence and pathogenicity of these rare serogroups are described less often, given their minimal impact on public health in comparison to the more prevalent serogroups. As a result, literature on their epidemiology remains limited [2]. Despite this, some studies suggest that patients with terminal complement pathway deficiencies exhibit higher susceptibility to both rare meningococcal serogroups (like serogroup E) and NG strains, being inherently defective in forming the terminal C5–9 membrane attack complex (MAC) on the bacterial surface and therefore complement-mediated bacterial clearance [3]. Additionally, recent advances in monoclonal antibody therapies targeting complement factor C5, such as eculizumab and, more recently, C3, have revolutionized the management of diseases caused by the dysregulation of the complement system like paroxysmal nocturnal hemoglobinuria (PNH) and atypical hemolytic uremic syndrome (aHUS). However, these treatments also induce acquired complement deficiencies by specifically inhibiting the terminal complement pathway, leaving treated patients vulnerable to infections from encapsulated bacterial pathogens, including rare meningococcal serogroups [4].

While current vaccines targeting serogroup B (4CMenB and fHbp bivalent vaccines) are also effective against other highly prevalent serogroups such as C, W, and Y [5], there is limited data regarding their efficacy against rare serogroups, such as serogroup E. Moreover, complement deficiencies (acquired and hereditary) impair bacterial lysis despite vaccination [6,7]. Given this uncertainty, further research is required to assess whether existing vaccination strategies provide adequate protection against these less common isolates.

Until 2022, vaccinations against serogroup B and against serogroup ACWY were not recommended in France for the general population. Only vaccination against serogroup C was recommended in the general population and became mandatory in 2018 for children under the age of 24 months [8]. These vaccines (B and ACWY) were only recommended for at-risk subjects and the control of outbreaks [9,10]. However, in 2024, meningococcal vaccination strategies in France were reevaluated and modified. Vaccination against MenB was recommended in France and reimbursed in 2022 among children 24 months of age and younger. It will be mandatory in January 2025 for the same age group. Vaccination against serogroups ACWY will also be mandatory for children 24 months of age and younger in January 2025, with a booster dose between 11 and 14 years of age and a catch-up to 24 years of age [11].

The present study aims to explore and describe the cases of IMD caused by rare (uncommon or unusual) serogroups other than A, B, C, W, and Y using the database of the National Reference Center for Meningococci and *Haemophilus influnezae* in France, covering 10 years from 2014 to 2023. This analysis will provide fresh insights into the epidemiology and genomic features of infections caused by these seldom-addressed serogroups, particularly in vulnerable and at-risk populations such as complement-deficient individuals.

## 2. Materials and Methods

### 2.1. Selection of the Study Cohort

In France, IMD is mandatorily reported. Epidemiological data on cases and serogroup distribution have been under close surveillance for several decades as part of the mission of our laboratory as a National Reference Center for Meningococci and *Haemophilus influnezae* (NRCMHi). We therefore retrospectively screened our database for all IMD cases (detection of *Nm* by culture and/or PCR from sterile sites) received between 2014 and 2023 and extracted epidemiological data (sex, age, and early mortality) and clinical data, including clinical manifestations as well as underlying comorbidities. The procedure for collecting samples and information was submitted and approved by the CNILN°1475242/2011 (Commission Nationale de l’Informatique et des Libertés), and the requirement for consent was waived.

### 2.2. Characterization of Clinical Isolates

IMD cases were biologically confirmed and grouped by culture and/or PCR. Full typing was performed using Whole Genome Sequencing by next-generation sequencing for cultured isolates. Primary samples underwent Sanger sequencing-based multilocus sequence typing (MLST). Genotypic data (ST and CC) were extracted using tools available on the PUBMLST.org databases. All sequences are available in this database by filtering on “country = France” and Period (>2013 and <2024).

### 2.3. Bactericidal Activity Assays and Prediction of Strain Vaccine Coverage

We conducted a serum bactericidal assay (SBA) using sera from subjects vaccinated with the 4CMenB vaccine and compared the bactericidal titers before and after vaccination against three isolates of serogroup E using human complement, as previously described [12]. Predictions of coverage by vaccines targeting group B isolates using the MenDeVar approach [13] were extracted using tools available in the PUBMLST.org databases. This method predicts vaccine coverage based on the alleles of genes encoding vaccine antigens present in a given isolate. Isolates were classified as “covered” if they had at least one allele encoding a covered peptide or as “non-covered” if all alleles encoded uncovered peptides. Isolates were classified as “unpredictable” if all the alleles encoded peptides for which available data were insufficient to predict coverage or non-coverage.

### 2.4. Measure of Complement Deposit on the Bacterial Surface

Isolates of each serogroup of *N. meningitidis* were grown overnight in a GC agar plate with supplements (Gibco, Thermo Fisher Scientific, Illkirch, France). A suspension containing 2 × 10^6^ CFU diluted in 50 µL of Hanks balanced salt solution supplemented with 0.15 mM calcium chloride and 0.5 mM magnesium chloride (HBSS^++^) (Gibco, Thermo Fisher Scientific, Illkirch, France) (HBSS^++^) with 1%BSA were mixed with 50 µL of normal sera from consent anonymous donors in a 96-wells plate and incubated for 30 min at 37 °C, 5% CO_2_. After the incubation, the bacteria were washed in HBSS^++^ with 1%BSA and then resuspended in a solution containing the FITC-conjugated anti-human C5b-C9 antibody (clone aE11 FITC conjugated antibody (HycultBiotech, Uden, The Netherlands)) diluted to 1:50 and incubated for 30 min in the dark at room temperature. Unstained bacteria served as negative control. After incubation, the bacteria were washed twice in HBSS^++^ with 1%BSA, resuspended with 2% paraformaldehyde (PFA) solution, and incubated for 30 min at room temperature in the dark. After incubation, the plate was centrifuged at 4000 rpm for 10 min and resuspended in 100 µL HBSS^++^ with 1%BSA. The plate was acquired using the CytoFlex S (Beckman Coulter, Villepinte, France), and the data were processed using FlowJo version 10.4.1 (BD Biosciences, East Rutherford, NJ, USA).

## 3. Results

### 3.1. Characteristics of IMD Cases of Unusual Serogroups

Screening of the NRCMHi database identified 3610 IMD cases for the period 2014–2023. Serogroup distribution of the 3610 IMD cases was as follows: 1784 (49.4%) were serogroup B, 627 (17.4%) were serogroup C, 575 (15.9%) were serogroup W, 565 (15.7%) were serogroup Y, and 59 (1.6%) were other serogroups (Table 1). These other serogroups included isolates of serogroups E, X and Z in addition to non-groupable isolates (all referred to hereafter as unusual groups). The IMD cases corresponding to these 59 isolates had a male/female ratio of 1.1, similar to the usual serogroups (1.0). The median age for these 59 IMD cases (21.5 years) was also comparable to that of the usual serogroup IMD cases (B, C, W, and Y) (22.3 years) (Table 1). It is noteworthy that serogroup B cases play a major role in explaining this median figure, as they account for half the number of cases of the usual serogroups, while the median ages for cases due to serogroups C, W, and Y were higher than those of the other serogroups (Table 1). The percentage of subjects under 18 years of age was not significantly lower among unusual serogroups and ranged between 0% and 43% versus 19% to 51% for serogroups B, C, W, and Y. Five cases due to unusual serogroups (four serogroup E isolates and one non-groupable isolate) reported vaccination against serogroups B and ACWY. Those five patients were all adults and were treated with anti-complement C5 monoclonal antibodies (Eculizumab or ravulizumab) for paroxysmal nocturnal hemoglobinuria (PNH) (see below in Section 3.2) [14,15]. Mortality was low among IMD cases due to unusual serogroups, with only one fatal case due to a serogroup X isolate (1.6%).

The 59 cases of unusual groups were confirmed by culture (*n* = 38, 64.4%), by PCR (*n* = 19; 32.2%) and in 2 cases by both culture and PCR (3.4%). Serogroup X was the most frequent (*n* = 21; 35.6%) among the capsulated isolates, followed by serogroup E (*n* = 11, 18.6%); one isolate (1.7%) was of serogroup Z, and 26 isolates (44.1%) were non-groupable. The overall percentage of the unusual groups was 1.6%, with a non-significant trend towards an increase in the last three years (*p* = 0.09). This percentage varied yearly from 0.5% (in 2014 and 2018) to 4.7% (in 2021). The distribution of clinical forms of IMD was also similar to that among IMD cases due to usual groups, mostly featuring bacteremia and/or meningitis. Cases associated with non-meningeal forms (pneumonia, abdominal presentation, or arthritis) were also present in 20% of the 59 cases. WGSs or MLST were only available for 54 cases (91.5%). Fifteen clonal complexes were identified among 46 isolates, while 8 isolates did not belong to any known clonal complex and were designated UA (UnAssigned). Isolates of clonal complex CC60 were the most frequent, and all belonged to serogroup E (*n* = 9; 16.7%), followed by isolates of CC1157 (*n* = 8; 14.8%) that were mainly of serogroup X (*n* = 7) with one E isolate. Notably, three isolates of CC181 (serogroup X) were also detected. Only four non-groupable isolates (7.4%) belonged to one of the hyperinvasive clonal complexes (2 from CC32 and 2 from CC269).

### 3.2. Complement Activation on Meningococcal Bacterial Surface

IMD cases that are due to unusual groups appear to be associated with complement deficiencies (hereditary or acquired deficiencies). Only 6 of the 59 cases (10.2%) reported in this study were associated with such complement deficiencies. One case was associated with late terminal complement pathway deficiency (TPD). The other five cases were associated with acquired deficiency due to treatment for Paroxysmal *nocturnal* hemoglobinuria (PNH) using monoclonal antibodies treatment (eculizumab or ravulizumab) against the C5 complement component (anti-C5 mAb). Of the six corresponding isolates, five belonged to group E, and one isolate was non-groupable (non-capsulated). One hypothesis is that the treatment with anti-C5 monoclonal antibodies impairs the formation of the MAC on the bacterial surface and therefore enhances the survival of such isolates. To explore this hypothesis, we tested the deposition of the terminal complement pathway components on bacterial surfaces using flow cytometry and a monoclonal antibody directed against the membrane attack complex C5b-C9 (MAC), as described in the Methods section.

**Figure 1:**
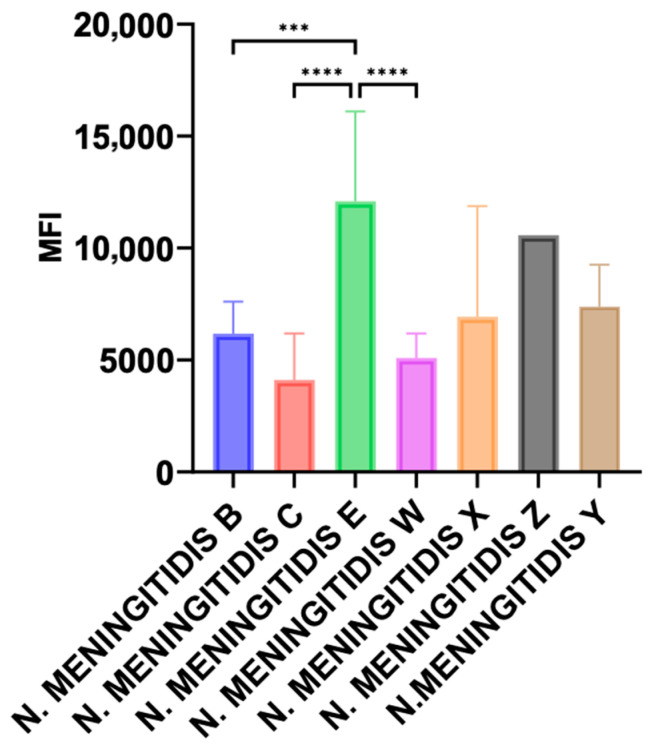
Flow cytometry analysis of deposition of membrane attack complex (MAC) at the surface of *N. meningitidis* isolates of several serogroups. Data are expressed as the mean of fluorescence index (MFI) for isolates of serogroups B (*n* = 16), C (*n* = 8), W (*n* = 14), E (*n* = 7), X (*n* = 6), Z (*n* = 1), and Y (*n* = 8). Significantly higher levels of MAC deposition were observed for isolates of serogroup E when compared to serogroups B, C, and W (*** *p* < 0.001 and **** *p* < 0.0001).

Isolates of the serogroups E (*n* = 7), X (*n* = 6) and Z (*n* = 1) from the current study were tested, and we added invasive isolates of serogroups B (*n* = 16), C (*n* = 8), W (*n* = 14) and Y (*n* = 8) belonging to hyperinvasive isolates. Data were expressed as the mean of fluorescence index for isolates of the same serogroup. Significantly higher levels of MAC deposition were observed for isolates of serogroup E when compared with serogroups B, C, and W (*p* < 0.001). Serogroup X isolates also showed higher levels compared with serogroups B, C, and W but did not reach statistical significance (Figure 1).

### 3.3. Coverage of Serogroup E Isolates by Vaccines Against Meningococci B

Data on vaccine coverage by both vaccines available against meningococci of serogroup B (4CMenB and the Bivalent fHbp) were extracted from WGS data for the 38 cases for which a cultured isolate was available. We first used the MenDeVar-based prediction that showed that 7/38 isolates (18.4%) were predicted to be covered by the 4CMenB, and 20/38 isolates (52.6%) were predicted to be covered by the Bivalent fHbp vaccine (Table 2) [13]. Serogroup E isolates were predicted to be covered by the serogroup B bivalent fHBP vaccine but were unpredictable for their coverage by the 4CMenB vaccine [13].

The use of the MenDeVar approach yielded many unpredictable isolates by the 4CMenB, most likely due to a high number of alleles of *fhbp* and *nhba* genes. We, therefore, used the phenotypic SBA approach and compared SBA titers in sera from subjects vaccinated by the 4CMenB vaccine before and after vaccination. SBA titers in sera before vaccination were below the threshold of 4, which correlates with protection but increased to 8 and 16 in sera after vaccination (above the threshold of 4, which correlates with protection) for the three serogroup E tested isolates. The results suggest that the 4CMenB vaccine covers these isolates.

## 4. Discussion

Our work highlights the unusual meningococcal serogroups that, although of low prevalence, require awareness as they may reveal or reflect special patient backgrounds, such as unusual presentation of IMD or association with complement deficiencies. Moreover, some isolates may be linked to travel/immigration, such as the serogroup X isolates of CC181 that were also reported in meningitis cases due to serogroup X in sub-Saharan Africa and refugee camps in Europe [16].

The variation in the number of IMD cases due to serogroup X from year to year is not clear. However, the number of cases is still too low (<10 cases per year) to allow meaningful statistical analysis. Surveillance needs to be strengthened to detect any further variation.

IMD cases due to these unusual serogroups were observed in all age groups, including children, adolescents, and young adults, as reflected by the median age of 21.5, which is similar to that of cases due to serogroup B. However, the percentages of children (under 18 years of age) were lower among cases due to unusual serogroups compared to serogroups B, C, W, and Y (29% versus 38%), but this difference was not significant. Cases due to serogroups W and Y are usually reported in adults or older adults [2], while IMD cases due to serogroup C have shifted to adults after the implementation of mandatory vaccination against meningococci of serogroup C in France since 2018 [17].

These unusual isolates usually do not belong to the major hyperinvasive genetic lineages. This observation is at odds with the percentage of invasive isolates belonging to the hyperinvasive clonal complexes (CC11, CC32, CC41/44, and CC269) in France, which was 66.8% before the COVID-19 pandemic [17]. We have previously reported that serogroup Y and non-groupable isolates were significantly more prevalent among TPD patients [3]. Serogroup E isolates were also associated with both acquired and hereditary complement deficiencies. Our data showed that serogroup E isolates exhibited the highest level of MAC deposition at the bacterial surface, which may prevent the invasion by these isolates in immunocompetent subjects. Capsular polysaccharide composition plays a key element in complement activation on the bacterial surface [18]. Serogroup B isolates were reported to be less permissive to complement activation and C3 deposition, while isolates of serogroup Y enhanced C3 activation and showed marked C3 deposition [19,20,21]. We reported here that isolates of serogroup E may behave similarly to isolates of serogroup Y by enhancing C3 deposition and stabilizing the C3 convertase, leading to a higher amount of MAC on the bacterial surface [22].

Subjects with terminal complement pathway deficiencies are reported to have repeated IMD but with a lower mortality rate than complement-proficient subjects [23,24]. Corroboratively, a low fatality was observed in our study, with a 1.6% fatality rate. Lower complement activation may lead to more favorable IMD outcomes in patients with late complement pathway deficiencies, including the use of anti-C5 treatments [18]. Indeed, inflammatory and autoimmune mechanisms are primarily implicated in mortality and morbidity observed in several pathological agents leading to endocrinological complications [25]. Such complications associated with massive adrenal hemorrhagic lesions and overwhelming sepsis were also observed in IMD with usual meningococcal serogroups as well as other bacterial species [26,27].

Unusual isolates do not belong to hyperinvasive genetic lineages of meningococci and are expected to show lower virulence [1,28]. Indeed, isolates of serogroups E, X and Z are frequently detected among carriers compared to IMD cases [29,30]. Moreover, when MAC is missing, the invasiveness of these bacteria is enhanced, and carriage isolates that are frequent [31] may acquire sustained invasiveness. These data agree with a higher susceptibility to IMD due to serogroup E isolates when MAC is missing, as in patients with TPD or those treated with anti-C5 mAb. Corroboratively, five cases reported here (four due to isolates of serogroup E and one non-groupable isolate) were from patients under treatment with anti-C5 for PNH. This treatment has also been reported to improve outcomes for patients with atypical hemolytic uraemic syndrome [32].

Other indications for the use of anti-C5 are increasingly reported, such as *myasthenia gravis, where Ravulizumab or* Eculizumab treatments were associated with improvements in activities of daily living in patients suffering from *myasthenia gravis* [33,34]. Neuromyelitis optica spectrum disorders (NMSOD) can also benefit from treatment with anti-C5 treatment [35].

Eculizumab and ravulizumab treatments increase the risk of IMD [4]. However, the impairment of the complement pathway in these subjects under unti-C5 treatment and, in particular, the impairment of the terminal complement pathway (C5b–C9) may not be rescued even with anti-meningococcal vaccination [7,36]. Failure of vaccination was reported with IMD cases in patients under anti-C5 treatment [37,38]. Adding antibiotic prophylaxis to vaccination is also recommended in subjects under anti-complement treatment, such as the use of anti-C5 monoclonal antibodies.

Our data suggest that cases due to unusual serogroups seem to increase in the last three years. However, it is less likely that anti-meningococcal vaccination strategies against serogroups B and ACWY, which were almost absent in the general population during the study period (2014–2023), have impacts on IMD cases due to unusual serogroups.

Vaccines targeting meningococci B are composed of proteins and may be effective against isolates of other serogroups when isolates match with vaccine components [5]. Our data further provide evidence that isolates of unusual serogroups can also be covered by vaccines that were licensed against isolates of serogroup B. This result supports the recommendation for a wide anti-meningococcal vaccination, including ACWY and B vaccines, among patients with complement deficiencies. The recently licensed pentavalent vaccines (ABCWY and ACWXY) can also be of interest in subjects with acquired and hereditary complement deficiencies [39,40,41]. Implementation of vaccines with expanded coverage to include these six serogroups (A, B, C, W, X, and Y), which are responsible for almost all IMD meningococcal, can be used to specifically target patients with complement deficiencies in addition to their household contacts [42].

The burden of cases due to isolates of unusual serogroups is expected to increase in proportion, and their number is also expected to rise upon the increasing indication of anti-complement drugs [43].

## 5. Conclusions

IMD cases due to the usual serogroups in Europe (B, C, Y, and W) are expected to decrease upon the implementation of vaccination strategies against these serogroups. Therefore, enhanced surveillance of cases due to other serogroups and analysis of the corresponding isolates is warranted.

## Figures and Tables

**Table 1:** The data depicting the distribution of IMD cases and the characteristics of the isolates and their distribution per serogroup over the period 2014–2023.

Year	B	C	W	Y	B, C, W, Y	Others	X	E ^¥^	Z	NG ^§^	All
2014	217	111	19	40	387	2	1	0	0	1	389
2015	213	110	27	51	401	9	1	2	0	6	410
2016	219	111	45	57	432	6	4	1	0	1	438
2017	190	121	69	73	453	3	2	0	0	1	456
2018	191	88	57	55	391	2	0	1	0	1	393
2019	210	51	87	51	399	10	8	1	0	1	409
2020	112	20	39	23	194	5	2	1	0	2	199
2021	66	3	16	16	101	5	2	0	0	3	106
2022	142	8	55	66	271	6	0	0	1	5	277
2023	224	4	161	133	522	11	1	5	0	5	533
2014–2023	1784	627	575	565	3551	59	21	11	1	26	3610
Age Median	17.7	27.8	40.6	53.4	22.3	21.5	21.5	21.1	19.7	23.3	22.3
% of under 18 years of age	51%	30%	27%	19%	38%	29%	43%	9%	0%	27%	37%
M/F sex ratio	1.1	1.0	0.8	0.8	1.0	1.1	0.3	2.7		1.9	1.0

Numbers of cases per year and per serogroup are shown. “all usual” stands for serogroups B, C, W, and Y) and “all unusual” stands for the other serogroups (X, E, Z, and non-groupable NG). ^¥^ Four IMD cases due to serogroup E isolates reported vaccination against serogroups B and ACWY and corresponded subjects under treatment with anti-complement C5 monoclonal antibodies (Eculizumab or ravulizumab) for paroxysmal *nocturnal* hemoglobinuria (PNH). ^§^ One IMD case due to NG isolates.

**Table 2:** Prediction of vaccine coverage by MenB vaccines of isolates of unusual serogroups.

	E (*n* = 8) *	X (*n* = 13)	Z (*n* = 1)	NG (*n* = 16)	All (*n* = 38)
	4CMenB vaccine				
Covered	0	3	0	4	7
Unpredictable	8	10	1	12	31
	Bivalent fHbp vaccine				
Covered	8	6	1	5	20
Unpredictable	0	7	0	11	18

* Total numbers of isolates of each serogroup (E, X, Z, and non-groupable, NG) are indicated. The Numbers of isolates that were predicted to be “covered” or “unpredictable” were shown for each vaccine.

## Data Availability

The original contributions presented in this study are included in the article. Further inquiries can be directed to the corresponding authors.
